# Weaned piglets: another factor to be considered for the control of *Salmonella* infection in breeding pig farms

**DOI:** 10.1186/s13567-019-0666-7

**Published:** 2019-06-18

**Authors:** Alejandro Casanova-Higes, Clara Mª Marín-Alcalá, Sara Andrés-Barranco, Alberto Cebollada-Solanas, Julio Alvarez, Raúl C. Mainar-Jaime

**Affiliations:** 1 Unidad de Producción y Sanidad Animal, Centro de Investigación y Tecnología Agroalimentaria de Aragón, Instituto Agroalimentario de Aragón-IA2-(CITA-Universidad de Zaragoza), Zaragoza, Spain; 2 Grupo de Genética de Micobacterias, Departamento de Microbiología, Medicina Preventiva y Salud Pública, Universidad de Zaragoza, Zaragoza, Spain; 3 Unidad de Biocomputación, Instituto Aragonés de Ciencias de la Salud (IACS/IIS Aragón), Centro de Investigación Biomédica de Aragón (CIBA), 50009 Zaragoza, Spain; 4 Centro de Vigilancia Sanitaria Veterinaria VISAVET, Universidad Complutense de Madrid, 28040 Madrid, Spain; 5 Departamento de Sanidad Animal, Facultad de Veterinaria, Universidad Complutense de Madrid, Madrid, Spain; 6 Departamento de Patología Animal, Facultad de Veterinaria, Instituto Agroalimentario de Aragón-IA2 (Universidad de Zaragoza-CITA), Zaragoza, Spain; 7 CIBER Enfermedades Respiratorias, Instituto de Salud Carlos III, Madrid, Spain

## Abstract

Field studies on *Salmonella* infection in suckling piglets are scarce due to the intrinsic difficulties of collecting proper samples (i.e. tonsils or mesenteric lymph nodes), and most of them rely on the analysis of rectal swabs that limit their accuracy. We used 495 slaughtered 4-weeks-old male piglets intended for human consumption from 5 *Salmonella*-seropositive breeding farms to collect gastrointestinal packages and perform a thorough detection of *Salmonella* on mesenteric lymph nodes and intestinal content. The overall prevalence of both infection and shedding was high (≈ 36%) indicating that piglets played an active role in *Salmonella* maintenance in the farms. Major serotypes found in piglets included 4,[5],12:i: (35.4%), Rissen (17.1%), Derby (10.9%) and Bovismorbificans (10.3%). In most of the infected animals (72.8%) the same serotype was found in mesenteric lymph nodes and feces. Significant higher ELISA OD% values were found in meat juice samples from non-infected piglets compared to infected ones (median OD% of 12.0 and 17.3, respectively; *P* = 0.002) suggesting some protective effect of sow’s colostrum. *Salmonella* was also isolated from feces from weaned sows contemporary of the slaughtered piglets, and 89% of the serotypes identified in sows were also detected in piglets. Pulsed field gel electrophoresis analyses showed that 75% of the piglet isolates that were compared to those of sows were related to them, suggesting the circulation of *Salmonella* strains between sows and piglets. It appears that improving piglet colostrum intake along with the reduction of the shedding in sows may favor the control of *Salmonella* infection in breeding farms.

## Introduction

Salmonellosis is the second most commonly reported bacterial foodborne infection in humans in the European Union (EU) after campylobacteriosis [1]. *Salmonella* spp. were the most frequently reported causative agents of food-borne and waterborne outbreaks in 2017, causing 24.4% of the outbreaks, which represented a moderate increase in EU compared to the 2014–2015 period. The consumption of contaminated pig meat and products thereof is considered one of the most important sources of human infection in *Salmonella* outbreaks in the EU [1]. Thus, Public Health authorities have advised on the need for control of *Salmonella* infection in swine, and several EU countries have initiated National Control Programs to reduce its prevalence in the pig population [2].

Pig meat production is a complex process that may be divided in several clearly defined periods: lactation (from the piglet birth to weaning at 3–4 weeks of age), nursery (from weaning to around 2.5 months old or 20–25 kg live weight), and the growing and fattening period (from 20 to 25 kg to 90–110 kg live weight). The dynamics of *Salmonella* infection has been extensively studied during the latter period [3–8], as fattening pigs are intended for human consumption and are considered the main source of *Salmonella* carcass contamination at slaughter [9, 10]. During this period there are multiple opportunities to collect samples either on farm (blood and feces) or at slaughter (blood, meat juice, gastrointestinal content, mesenteric lymph nodes, tonsils or the carcasses). Altogether, this allows for a proper monitoring of *Salmonella* infection dynamics at this stage and therefore the implementation of suitable on-farm interventions. Thus, most control programs have focused on this period of fattening, and different interventions have been implemented with more or less success to reduce the prevalence of infection on the farm [11, 12].

In contrast, there is an important lack of information on the epidemiology of *Salmonella* infection in the previous production phases, i.e. lactation and nursery even though it may influence the dynamics of infection during the growing and fattening period [13]. During nursery, for example, piglets are highly vulnerable to enteric pathogens, such as *Salmonella* spp. [14, 15]. Bacterial colonization by these pathogens is favored by the intestinal dysbiosis commonly observed in weaned piglets after diet change (from milk-based feed to gross feed) and the stress associated with new environments and the comingling of pigs [16, 17]. However, *Salmonella* infection and shedding is probably overlooked at this stage, mostly due to the common use of in-feed antimicrobials (i.e. colistin, Zinc oxide) that hinders the detection of pathogenic enteric Gram negative bacteria. In addition, proper field studies assessing the prevalence of *Salmonella* infection in nursery pigs would be expensive, as they would require the unethical slaughter of a large number of young animals.

With regard to suckling piglets in intensive-reared pig farms, a few published studies suggest that the prevalence of *Salmonella* shedding is, in general, low (< 10%) [3, 17–20]. Even when pools of fresh feces were used, the mean prevalence of shedding in these piglets was < 5% [21]. However, results from all these studies are based mostly on the analysis of a small amount of feces, in many occasions collected through rectal swabs. Sensitivity of bacteriology from fecal samples is known to be directly related to the amount of feces used for analysis [22–24], and thus these studies may have underestimated the true prevalence of *Salmonella* shedding. Besides, lack of *Salmonella* shedding does not necessarily prove absence of infection, as intermittent shedding has been observed in *Salmonella*-infected pigs [3, 25–27]. Since suckling piglets may act as a significant source of *Salmonella* for subsequent production phases, accurate information on the dynamics of *Salmonella* infection at the end of the lactation period would be of much interest for implementing preventive interventions at this stage.

Roasted piglet (the so-called “cochinillo asado” in Spanish) is a widespread delicatessen food consumed all over Spain. In 2016, more than 1.8 million weaned piglets were slaughtered for human consumption in specialized commercial abattoirs [28]. Thus, for the present study, we took advantage of the availability of these type of abattoirs to assess in a more accurate way the prevalence of *Salmonella* infection and shedding in a population of weaned piglets coming from breeding farms where *Salmonella* was circulating. We also evaluated the serological status of these animals with regard to *Salmonella* specific antibodies to better understand the role that their presence may have on *Salmonella* infection at this age. All these results should help to shed more light on the risk factors for transmission of this infection within infected breeding farms and design better methods for its control.

## Materials and methods

### Farm selection and collection of samples

Five (A, B, C, D and E) multiplier/supplier *Salmonella*-seropositive breeding farms from the Northeast part of Spain (one of the largest pig-production areas in Europe) and that showed their willingness to collaborate were included. Farm size ranged from 700 to a maximum of 940 sows. Sampling was carried out in two periods, between February 2012 and July 2013 (farms A, B and C), and March 2015 and April 2016 (D and E). In these breeding farms, female weaned piglets were reared as re-stocking gilts for pig production farms, while male weaned piglets were sent to the abattoir for meat. The males were slaughtered when they reached ≥ 7 kg live weight, which usually occurred at weaning (≈ 4 weeks old -4 wo-). Thus, all the samples analyzed in this study belonged to male piglets of this age.

Piglet samplings were carried out along the year in one abattoir and they depended upon both piglet availability from the five selected farms and abattoir staff readiness for collaboration. The whole piglet intestinal packages were collected directly from the slaughter line every time that a sampling was scheduled. Samples were then submitted to the laboratory for immediate processing. From each package the maximum possible amount of mesenteric lymph nodes (MLN) and as much intestinal content (IC) as possible (from the cecum to the rectum) were collected for bacteriological analysis. A piece of the diaphragm muscle was also collected for serological analysis.

To determine the most prevalent serotypes circulating in the farms, every 3–4 months during the period of piglet sampling, farm staff collected fecal samples directly from the rectum of 10–12 recently weaned sows. These sows were not directly related (i.e. dams) to the studied piglets, but were present in the farm at the same time that piglets were sent to slaughter.

In addition, serum samples from ≈ 120 sows (minimum 116, maximum 158) in each farm were available. They had been routinely collected every 3–4 months during the period of piglet sampling and within the frame of the official eradication campaign for Aujeszky’s disease. These sows were not necessarily related to the piglets analyzed either, but they were used to assess the *Salmonella* serological status in the 5 farms.

### Bacteriology

Bacteriology on both IC and MLN samples was performed according to the EN ISO 6579:2002/A1:2007 [29]. Fresh MLN samples were first defatted, weighed, and externally decontaminated by dipping into absolute alcohol and further flaming. Afterwards, samples were homogenized in buffered peptone water -BPW- (Panreac Quıímica SAU, Castellar del Vallés, Spain) in 1:10 dilution and incubated for 18 ± 2 h at 37 ± 1 °C. Thereafter, 3 drops (33 µL each) of incubated BPW were inoculated into a modified semisolid Rappaport–Vassiliadis (MSRV, Oxoid Ltd., Hants, UK) medium, and plates were incubated for 24 ± 3 h at 41.5 ± 1 °C (negative samples were reincubated for an additional 24 h). One microliter of the presumptive *Salmonella* growth (detected by the halo generated in MSRV after 24 or 48 h) was transferred to two selective media (xylosine lysine deoxycholate [XLD] and brilliant green [BG] agars (Panreac Quıímica SAU). Suspected colonies were confirmed biochemically (triple sugar iron [TSI] agar, urea agar, l-lysine decarboxylation medium, and indole reaction (Panreac Quıímica SAU). One colony of *Salmonella* spp. per plate from each *Salmonella*-positive MLN and IC sample was further serotyped at the National Reference Laboratory for Animal Salmonellosis -NRLAS- (Madrid, Spain) following the White–Kauffmann–Le Minor scheme [30].

### Pulsed-field gel electrophoresis analysis

To assess the genetic relationship between *Salmonella* infection (i.e. MLN+) and *Salmonella* shedding (IC+) for a given piglet, and between *Salmonella* infection in piglets and *Salmonella* shedding in sows, pulsed-field gel electrophoresis (PFGE) analysis was performed on *Salmonella* isolates according to the Pulse-Net protocol [31] as described in detail by [32]. Due to budget restrictions, not all *Salmonella* isolates were analyzed by PFGE. Only isolates meeting the following criteria were considered for analysis:

1. For the assessment of the relationship of *Salmonella* infection and shedding in piglets: when the same *Salmonella* serotype was isolated from MLN and IC samples from the same piglet, then these two isolates were analyzed by PFGE analysis. If this occurred in several piglets from the same farm and within the same batch, a maximum of two piglets per batch were analyzed.
2. For the assessment of the relationship between *Salmonella* infection in piglets and *Salmonella* shedding in weaned sows: when the same *Salmonella* serotype was isolated from a piglet's MLN sample and from a fecal sample from any of the sows analyzed from the same farm. A maximum of two piglet isolates and two sow isolates per batch were analyzed.

PFGE pattern analysis was performed with the BIONUMERICS software (version 6; Applied Maths, Sint-Martens-Latem, Belgium) using Dice coefficient and unweighted pair group method with arithmetic averages (UPGMA dendrogram type) with a position tolerance of 1.5% and optimization of 2.0%.

### Serology

Diaphragm muscle samples were frozen and thawed to obtain meat juice (MJ). Sow serum samples and piglet MJ were kept at −20 °C until their use. To detect specific antibodies (IgG) against *Salmonella* spp. in both type of samples, the Herdcheck Swine *Salmonella* ELISA test (IDEXX Laboratories, Westbrook, ME, USA) was used following manufacturer’s instructions. This test targets the main swine *Salmonella* serogroups (B, C1 and D). For piglets, results were presented as optical density percentage (OD%) values. In case sows, a cutoff-value of OD% ≥ 40 was considered to classify a sow as seropositive. This threshold was selected given the low specificity of the ELISA test used [33, 34].

### Statistical analyses

Piglet prevalence of infection and shedding and their corresponding 95% confidence intervals (95% CI) were estimated. The weight of MLN and IC samples was compared between the corresponding *Salmonella*-positive and *Salmonella*-negative piglets for each type of sample by means of the Mann–Whitney test for independent samples to detect potential effects on bacteriological results. The relationship between piglet shedding and infection was assessed by mixed logistic regression after adjusting by season and considering farm as a grouping factor (gllamm module in STATA). ELISA OD% values between *Salmonella*-infected and non-infected piglets were compared using the Kruskal–Wallis test. The software STATA (STATA/IC 12.1. Stata-Corp. LP, College Station, TX, USA) was used for all statistical analyses.

## Results

### *Salmonella* isolation, serotyping and serology in piglets

A total of 495 MLN and 495 IC samples were analysed from the corresponding 495 weaned piglets (a mean of 99 piglets per farm). Piglets were sampled in all seasons but autumn due to abattoir availability. The distribution of the sampling by farm and their corresponding prevalences of infection (MLN+) and shedding (IC+) are shown in Table 1. The overall prevalence of infection varied significantly between farms, ranging from 17.8 to 70.4% with an overall value of 36.0% (95% CI 31.9, 40.3). The prevalence of shedding piglets also varied significantly between farms, with a similar overall value (35.4%; 95% CI 31.3, 39.7).

**Table 1:** **Results for** ***Salmonella*** **isolation** ^**a**^ **from intestinal content and mesenteric lymph nodes in 4-weeks-old piglets**

Farm	No. of piglets	No. of IC + (%)	No. of MLN + (%)	No. of IC + and MLN + (%)^b^
A	105	30 (28.6)	19 (18.1)	15 (44.1)
B	118	16 (13.6)	21 (17.8)	6 (19.4)
C	99	36 (36.4)	29 (29.3)	20 (44.4)
D	92	42 (45.7)	52 (56.5)	38 (67.9)
E	81	51 (63.0)	57 (70.4)	46 (74.2)
Total	495	175 (35.4)	178 (36.0)	125 (54.8)

IC: intestinal content, MLN: mesenteric lymph nodes.

^a^ISO 6579:2002/Amd 1:2007.

^b^Percentage estimated from positive (either IC or MLN) piglets.

A median of 7.6 g (95% CI 7.4–7.9) of MLN and of 27 g (95% CI 26.5–27.6) of IC was collected. No significant differences in weights between IC-positive and IC-negative samples were observed (median of 26.8 g and 27.1 g, respectively; *P* = 0.60). However, the weight for MLN-positive samples was significantly higher than that for MLN-negative samples (median of 8.2 g and 7.3 g, respectively; *P* < 0.001).

All *Salmonella* isolates (175 from IC samples and 178 from MLN samples) were serotyped. The monophasic variant of *S*. Typhimurium (*S*. 4,[5],12:i:) was the most frequent serotype (35.4%) recovered from IC samples, followed by Rissen (17.1%), Derby (10.9%) and Bovismorbificans (10.3%). *Salmonella* 4,[5],12:i: was also the most frequent serotype (27.5%) in MLN samples, followed by Bovismorbificans (20.2%), Rissen (14%), and Derby (11.8%). Both 4,[5],12:i: and Derby were present in all the farms and in both type of samples. The distribution of the *Salmonella* serotypes by type of sample is shown in Table 2.

**Table 2:** **Distribution of the** ***Salmonella*** **serotypes in piglets and sows isolates among the 5 farms**

Farm	Piglets isolates				No. of piglets with the same serotype in MLN-IC	Sows fecal isolates	
	IC		MLN				
	Serotype	No. (%)	Serotype	No. (%)		Serotype	No. (%)
A	4,[5],12:i:-	12 (40)	4,[5],12:i:-	8 (42)	4	Kapemba	11 (68.7)
	Muenchen	5 (16.7)	Muenchen	3 (15.8)	1	4,[5],12:i:-	4 (25)
	Kapemba	5 (16.7)	Kapemba	2 (10.5)	1	Rissen	1 (6.3)
	Rissen	5 (16.7)	Rissen	2 (10.5)	1		
	Derby	2 (6.6)	Derby	2 (10.5)	1		
	Bredeney	1 (3.3)	Typhimurium	2 (10.5)			
B	Derby	7 (43.8)	Derby	8 (38.1)	6	Brandenburg	8 (88.9)
	Typhimurium	4 (25)	Typhimurium	2 (9.5)		Goldcoast	1 (11.1)
	Anatum	2 (12.5)	Anatum	1 (4.8)			
	4,[5],12:i:-	1 (6.3)	4,[5],12:i:-	2 (9.5)			
	Brandenburg	1 (6.3)	Brandenburg	2 (9.5)			
	Rissen	1 (6.3)	Rissen	5 (23.1)			
			Infantis	1 (4.8)			
C	Rissen	23 (63.9)	Rissen	17 (58.6)	14	Anatum	7 (53.8)
	Infantis	4 (11.1)				Bovismorbificans	4 (30.8)
	4,[5],12:i:-	2 (5.5)	4,[5],12:i:-	5 (17.2)	1	London	1 (7.7)
	Anatum	2 (5.5)	Anatum	1 (3.5)	1	Typhimurium	1 (7.7)
	Derby	2 (5.5)	Derby	2 (6.9)			
	London	2 (5.5)	Bovismorbificans	1 (3.5)			
	Agona	1 (2.2)	Agona	3 (10.3)	1		
D	4,[5],12:i:-	34 (81)	4,[5],12:i:-	22 (42.3)	17	Anatum	1 (100)
	Bovismorbificans	4 (9.5)	Bovismorbificans	21 (40.4)	4		
	Derby	3 (7.1)	Derby	2 (3.8)	2		
	Brandenburg	1 (2.4)	Brandenburg	4 (7.7)			
			Rissen	1 (1.9)			
			Typhimurium	1 (1.9)			
			Muenchen	1 (1.9)			
E	Bovismorbificans	14 (27.5)	Bovismorbificans	14 (24.6)	11	Anatum	7 (87.5)
	4,[5],12:i:-	13 (25.5)	4,[5],12:i:-	12 (21.1)	9	Kapemba	1 (12.5)
	Anatum	9 (17.6)	Anatum	12 (21.1)	9		
	Derby	5 (9.8)	Derby	7 (12.3)	3		
	Ohio	3 (5.9)	Ohio	3 (5.3)	3		
	Panama	3 (5.9)	Panama	6 (10.5)	1		
	Infantis	2 (3.9)	Infantis	2 (3.6)	1		
	Rissen	1 (2)	London	1 (1.8)			
	Typhimurium	1 (2)					
Total		175		178	91		47

*Salmonella* was not detected in 267 (53.9%) of the sampled piglets, while positive results in both MLN and IC samples were obtained for 125 (25.2%) of them. Of these, 91 (72.8%) showed the same serotype in both samples (Table 2). A significant association between the isolation of *Salmonella* in MLN and IC samples was observed: a MLN-positive piglet had around 10 times higher odds of shedding *Salmonella* than a MLN-negative piglet (OR = 10.27; CI 6.31–16.86; *P* < 0.001) once the season and farm effects were accounted for (Table 3).

**Table 3:** **Association between** ***Salmonella*** **infection and** ***Salmonella*** **shedding in weaned piglets by mixed logistic regression analysis**

	No. of piglets	No (%) of IC + piglets	Logistic regression parameters		
			OR	95% CI (OR)	*P*
MLN					
Negative	320	53 (16.6)	1		
Positive	175	125 (71.4)	10.27	6.31, 16.86	< 0.001
Season					
Winter	176	99 (56.3)	1		
Spring	222	48 (21.6)	0.35	0.21, 0.58	< 0.001
Summer	97	28 (28.9)	0.38	0.19, 0.75	0.006

Farm used as grouping factor.

IC: intestinal content, MLN: mesenteric lymph nodes, OR: odds ratio.

Overall, the median OD% value in all 495 animals was 15.9 (median 95% CI 13.7–17.8). Significantly higher OD% values in MLN-negative piglets were observed compared to the MLN-positive ones (median of 17.3 vs. 12.0, respectively; *P* = 0.002). Similar results were found for IC-negative piglets compared to IC-positive piglets (17.2 vs. 12.3, respectively; *P* = 0.016).

### *Salmonella* isolation, serotyping and serology in sows

A total of 214 fecal samples from weaned sows were collected. The overall prevalence of *Salmonella* shedding among those was 21.9% (95% IC 16.9–27.9), but it varied significantly between farms, ranging from 2.1 to 40% (Table 4). The serotypes found in sows differed among farms (Table 2). The most frequent serotype was Anatum (31.9%) which was present in 3 farms (C, D and E), followed by Kapemba (25.5%; farms A and E), and Brandenburg (17%; farm B). In general, the serotypes most commonly detected in sows were also detected in piglets from the same farm (Table 2).

**Table 4:** **Results for** ***Salmonella*** **isolation** ^**a**^ **and** ***Salmonella*** **seroprevalence** ^**b**^ **in weaned sows from 5 farms**

Farm	No. of fecal samples	No. of + fecal samples (%)	No. of serum samples (%)	No. seropositive samples (%)^b^
A	40	16 (40)	144	82 (56.9)
B	40	9 (22.5)	134	115 (85.8)
C	40	13 (32.5)	134	98 (73.1)
D	47	1 (2.1)	158	129 (81.6)
E	47	8 (17)	116	70 (60.3)
Total	214	47 (21.9)	686	494 (72)

^a^ISO 6579:2002/Amd 1:2007.

^b^Considering a cut-off value OD% ≥ 40% (Herdcheck Swine *Salmonella* ELISA test, IDEXX Laboratories, USA).

Six hundred and eighty-six sow serum samples were available from the official eradication campaign for Aujeszky’s disease. The overall seroprevalence was 72% (95% CI 68.5–75.2). Seroprevalence varied among farms, but it was always higher than 50% in all of them (Table 4).

### PFGE

One hundred and nine *Salmonella* isolates from piglets met any of the criteria described above for performing PFGE analysis. Twenty-two of them were not included because they belonged to serotypes that could not be typed by this technique (i.e. Panama, Ohio and Kapemba). Thus, 87 (24.6%) piglet isolates (47 MLN and 40 IC) out of 353 *Salmonella* isolates were analyzed by PFGE. Regarding sows, PFGE was performed on 19 (40.4%) out of the 47 *Salmonella* isolates.

**Figure 1:**
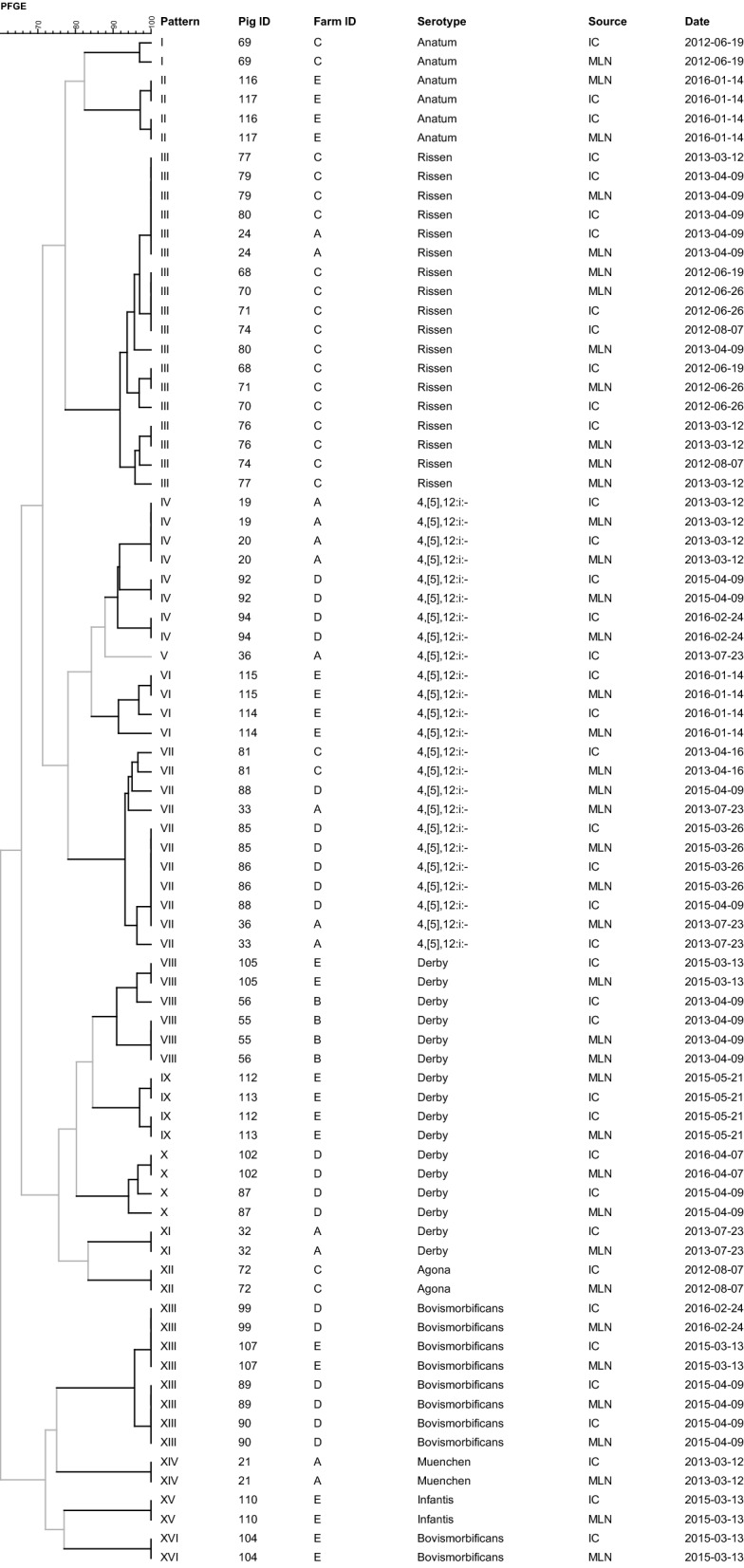
**Dendrogram showing PFGE patterns (≥ 90% homology) for 80** ***Salmonella* isolates from 40 piglets.** IC, intestinal content; MLN, mesenteric lymph nodes.

Forty piglets showing the same *Salmonella* serotype in both MLN and IC samples were selected for PFGE analysis. Sixteen different PFGE patterns were identified among the 80 isolates (Figure 1). In 97.5% (39) of the piglets, a PFGE homology ≥ 90% was found between *Salmonella* isolates from MLN and IC samples. In 22 (56.4%) of them a perfect match (100% homology) was observed.

**Figure 2:**
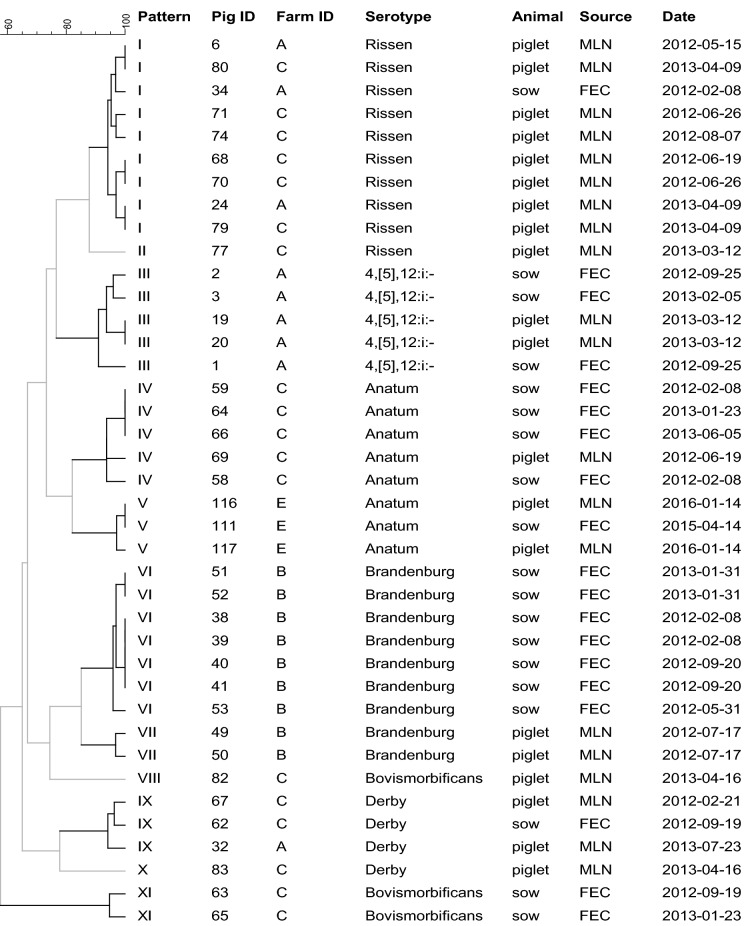
**Dendrogram showing PFGE patterns (≥ 90% homology) for 19** ***Salmonella* isolates from sows and 20 from piglets.** FEC, fecal sample; MLN, mesenteric lymph nodes.

In piglets from 4 farms (A, B, C and E) the genetic relationship between piglet infection and sow shedding could be assessed (the same serotype found in sow samples was found in at least one of the piglets from the same farm—Table 2). Nineteen *Salmonella* isolates from sows and 20 isolates from piglets were compared by PFGE. The sow isolates were grouped into 7 different PFGE patterns (> 90% genetic homology), and in 5 of them isolates from piglets were included (patterns I, III, IV, V, and IX—Figure 2). These 5 clusters comprised 75% of the piglet isolates analyzed.

Results from dendrograms also allowed identifying long-term patterns of infection (Figures 1, 2). Major piglets’ serotypes showing ≥ 90% PFGE homology were detected in several occasions within the same farm and sometimes with more than 200 days of difference (i.e. in farm A: Rissen; in B: Brandenburg; in C: Derby and Rissen; and in D: Bovismorbificans, Derby and 4,[5],12:i:-). Likewise, homologous *Salmonella* strains coming from piglets and sows were isolated more than 200 days apart in farm A (4,[5],12:i:-), C (Derby and Anatum), and E (Anatum). In addition, in farms A and B homologous *Salmonella* strains from sows were isolated more than 300 days apart (4,[5],12:i:- and Brandenburg, respectively).

## Discussion

To the authors’ knowledge, this is the first field study aiming at assessing the dynamics of *Salmonella* infection in slaughtered weaned piglets. These piglets were slaughtered for human consumption and were therefore considered clinically healthy and had not received any recent antibiotic treatment. These circumstances may have favored the detection of subclinically *Salmonella*-infected piglets. At slaughter, whole intestinal packages were collected and a thorough bacteriological study was carried out in order to detect *Salmonella* from both MLN and IC samples for a better assessment of the true prevalence of *Salmonella* infection and shedding at weaning, a pig production time scarcely studied [13]. Piglets belonged to farms where the mean within-herd sow seroprevalence remained high (≥ 50%) throughout the study, suggesting an active circulation of *Salmonella* while piglets were being weaned.

The overall proportion of *Salmonella* shedders in this population of weaned piglets was unexpectedly high (35.4%), although variable among farms (Table 1). It differed from previous studies on breeding herds that presented levels of sow seroprevalence or prevalence of shedding similar to this one, but that showed a much lower proportion of shedding piglets at this age, i.e. a range of prevalence from 0 to 9% [3, 17–19]. Considering the amount of intestinal content analyzed (an average > 25 g per piglet), the sensitivity of bacteriology in this study may have been maximized by the use of a larger amount of IC [22–24]. This may help to explain, at least in part, the overall higher prevalence of shedding observed when compared to these previous studies, which may have likely underestimated the true level of *Salmonella* shedding due to the sampling method used (mostly rectal swabs) in those suckling piglets. Other potential factor influencing these results may be related to the fact these animals were slaughtered and shedding was surely exacerbated by the stress associated with the transport and lairage [9, 26], making *Salmonella* detection more likely.

No previous surveys on prevalence of *Salmonella* infection were available at weaning. The overall prevalence in MLN was also strikingly high (36%) and virtually identical to that of shedding (Table 1). As many MLN as possible were collected from each piglet, amounting to an average of 8.1 g of MLN per animal. Given the size of MLNs at this age, this represents a substantial number of MLN that covered a large intestinal area (from the small to the large intestine), thus increasing the likelihood of detecting infected animals. This prevalence of infection may have been higher in case tonsils would have been analyzed, as *Salmonella* seems to persist long periods in tonsils [26], however this type of sample was not available from the abattoir.

Within each farm, the prevalence of *Salmonella* shedding and infection also seemed to match (Table 1), suggesting a likely relationship between infection and shedding in these piglets. In fact, the odds of shedding *Salmonella* at weaning was 10 times higher (OR = 10.27; Table 3) for MLN-positive piglets than that for MLN-negative piglets. A small proportion (16.6%) of the MLN-negative piglets shed *Salmonella*. This result was somewhat expected since up to 26% of the MLN-negative fattening pigs sent to slaughter may shed *Salmonella* [8], and shedding would be likely associated with recent infections from contaminated environments during transport and/or lairage. Experimental infections with *Salmonella* have shown that pigs shed *Salmonella* quickly after primary infection during approximately two weeks, after which they become intermittent shedders [3, 4, 25–27]. Given the age of these piglets (4 wo), it seems reasonable that most of these shedding piglets had been infected for first time during lactation. In 97.5% (39/40) of the piglets in which isolates of the same serotype were recovered from MLN and IC, a high genetic homology (≥ 90%) between them was found, and in 55% of them (22/40) a 100% match was detected (Figure 1). These results confirmed these weaned piglets acted as carriers of infection. The fact that they did not show clinical signs of disease emphasizes their potential role in the maintenance of *Salmonella* infection within these breeding farms. Thus, the presence of *Salmonella* in weaned pigs should be considered a potential risk factor for infection for pigs raised for slaughter. Strategies to prevent *Salmonella* infection during lactation and its further transmission to nursery will be required.

The monophasic variant of *S*. Typhimurium (4,[5],12:i:-) was the most common serotype in piglets (31.4%), followed by Rissen (15.6%), Bovismorbificans (15.3%) and Derby (11.3%). This distribution did not match that of sows, in which Anatum (31.9%) and Kapemba (25.5%) were the most frequently found serotypes, followed by 4,[5],12:i:- and Bovismorbificans (8.5% both). This discrepancy may be related to the fact that only one colony from each positive sample was serotyped, but animals could have been infected by different *Salmonella* serotypes at the same time [35]. In addition, it might be the result of differences in susceptibility to different *Salmonella* serotypes between young and adult pigs [27, 36–38].

The ELISA test used for serology on meat juice samples from piglets can detect specific immunoglobulins (IgG) against the main *Salmonella* serotypes found in pigs. Serological results showed significantly lower OD% values in *Salmonella*-infected compared to non-infected piglets (median OD% of 12.0 and 17.3, respectively; *P* = 0.002). Seropositivity in all weaned pigs was anticipated after the suckling of sow’s colostrum [39]. Maternally derived IgGs were expected to decrease gradually after birth, but then they would increase at 7 weeks of age due to the novo synthesis of immunoglobulins [40]. Thus, in these 4-wo piglets, the IgGs detected by the ELISA were most likely derived from sow’s colostrum [41]. The fact that OD% values were higher in non-infected piglets suggests some protective effect of the colostrum against *Salmonella* infection at this early age. Indeed, there are evidences from field studies showing that colostrum may be a critical factor to prevent *Salmonella* infection in piglets [42], and some experimental studies have shown that suckling pigs with higher antibody titres improved their resistance when challenged with *Salmonella* [43, 44]. Ensuring proper colostrum intake within the first hours of life should be a basic strategy to prevent *Salmonella* infection during lactation. Increasing the quality of colostrum (i.e. the amount of immunoglobulins) through vaccination of pregnant sows before farrowing should be considered another potential strategy to protect suckling piglets from infection [43, 45–47] and even *Salmonella* shedding in older pigs [48, 49].

Due to the stress associated with weaning, the post-weaning period seems to present higher risk for *Salmonella* shedding in sows [21, 50]. For this reason, recently weaned sows were sampled to determine the most prevalent on-farm circulating serotypes. Both the prevalence of shedding in weaned sows and the serotypes found were variable between farms (Tables 2 and 4). Overall prevalence of *Salmonella* shedding was higher (21.9%) than that reported in other studies with levels of seroprevalence similar to those found here [4, 17, 21, 50, 51]. This difference may be attributed to methodological differences between studies, such as including sows of different parities, animal management and immune status.

In agreement with previous studies, the distribution of *Salmonella* serotypes in these sows differed from those commonly isolated from finishing pigs [17, 52–54]. We also found some different serotypes within the farms between sows and piglets. However, 89% of the serotypes detected in the sows were also found in piglets from the corresponding farm. In addition, despite the low number of isolates from sows (19) and piglets (20) that were compared by PFGE, 75% of the piglet isolates were grouped within a PFGE pattern that included at least one *Salmonella* isolate coming from sows. Altogether this suggests that, in these farms, *Salmonella* infection can be maintained between sows and piglets. The fact that some *Salmonella* clones were detected in the farms over long periods (> 200 days) supports this hypothesis. Avoiding *Salmonella* shedding in sows seems to be important to prevent farm environmental contamination [21] and the subsequent infection of suckling piglets, which would end up shedding this pathogen later. The use of feeding strategies of proven efficacy in reducing *Salmonella* shedding on slaughter pigs such as fermented feed, some type of organic acids or some prebiotics [55–58] may help to control the infection in sows as well.

In conclusion, prevalence of *Salmonella* infection in weaned piglets from *Salmonella*-positive breeding herds may be much higher than previously reported. This study shows that suckling piglets can become subclinically infected and act as active carriers of *Salmonella*. There was a close relationship between *Salmonella* infection in piglets and sows as the same serotypes and strains were found in both populations. Colostrum intake may be a key factor to reduce the likelihood of piglet infection during lactation, but other on-farm strategies to reduce *Salmonella* shedding in sows are of utmost importance as well.

## Data Availability

The datasets supporting the conclusions of this article is available in the Research Gate Repository. 10.13140/RG.2.2.17677.79840.

## Abbreviations

4 wo: 4 weeks old
BG: brilliant green agar
CI: confidence interval
EU: European Union
FEC: fecal sample
IC: intestinal content
IgG: immunoglobulin G
MJ: meat juice
MLN: mesenteric lymph nodes
MSRV: modified semisolid Rappaport–Vassiliadis agar
OD%: optical density percentage
OR: odds ratio
PFGE: pulsed-field gel electrophoresis
TSI: triple sugar iron agar
XLD: xylosine lysine deoxycholate agar
